# The contribution of risk perception and social norms to reported preventive behaviour against selected vector-borne diseases in Guyana

**DOI:** 10.1038/s41598-023-43991-1

**Published:** 2023-10-06

**Authors:** Iris Lopes-Rafegas, Horace Cox, Toni Mora, Elisa Sicuri

**Affiliations:** 1 ISGlobal, Hospital Clínic Universitat de Barcelona, Barcelona, Spain; 2 Vector Control Services, Ministry of Health, Georgetown, Guyana; 3 Research Institute for Evaluation and Public Policies (IRAPP), Universitat Internacional de Catalunya (UIC), Barcelona, Spain; 4 LSE Health, London School of Economics and Political Science, London, UK

**Keywords:** Public health, Infectious diseases, Malaria

## Abstract

Preventing vector-borne diseases (VBDs) mainly relies on effective vector control tools and strategies, which in turn depend on population acceptance and adherence. Inspired by the abundant recent literature on SARS-COV-2, we investigate the relationship between risk perception and preventive behaviour for selected VBDs and the extent to which risk perception is determined by social norms. We use cross-sectional data collected from 497 individuals in four regions of Guyana in 2017. We use a conditional mixed process estimator with multilevel coefficients, estimated through a Generalized Linear Model (GLM) framework, applying a simultaneous equation structure. We find robust results on malaria: risk perception was significantly influenced by the risk perception of the reference group across different definitions of the reference group, hinting at the existence of social norms. Risk perception significantly increased the likelihood of passive behaviour by 4.48%. Less clear-cut results were found for dengue. This study applies quantitative social science methods to public health issues in the context of VBDs. Our findings point to the relevance of tailoring communications on health risks for VBDs to groups defined at the intersection of socio-economic and demographic characteristics. Such tailored strategies are expected to align risk perception among reference groups and boost preventive behaviour.

## Introduction

Vector-borne diseases (VBDs) account for more than 17% of all infectious diseases worldwide, causing more than 700,000 deaths annually (WHO). Despite being endemic to many areas of the globe, the morbidity and mortality burden associated with VBDs is disproportionately concentrated in low- and middle-income countries. VBDs constitute a significant threat among the poorest^1^. Due to climate change and the movement of people, the burden of disease associated with VBDs is expected to increase in endemic areas and expand geographically^2,3^. As a matter of fact, the World Health Organization has recently warned about the expansion of arboviruses in the American region well beyond the historical transmission areas^4^.

The prevention of VBDs relies on a few pharmacological measures, such as preventive chemotherapy and a series of vector control tools and strategies that either control the proliferation of vectors or avoid contact between humans and vectors (bed nets, indoor residual spraying, house improvement, skin repellents, coils, stagnant water management, etc.). Notably, most VBDs are not yet vaccine-preventable. The effectiveness of the available tools and strategies depends on individuals’ “preventive behaviour”, that is, on the acceptance of and adherence to the existing measures.

The SARS-COV-2 pandemic has re-focused the attention of researchers on the relationship between preventive behaviour and risk perception^5–9^. Although risk perception has several definitions, it is typically defined as the subjective probability of the occurrence of an adverse event in the future and the expected magnitude of its consequences^10^. Risk perception refers, therefore, to uncertain future events^11^. For this reason, preventive behaviours against uncertain adverse health events are conditioned to risk perception^12–15^.

In the context of SARS-COV-2, preventive behaviour refers to a series of measures such as vaccination, mask-wearing, hand washing, and “social” distancing. It has been proven that adherence to these measures depends on how high people perceive the risk of infection or, even more impactfully, the risk of severe disease or death^16–18^. Noticeably, risk perception has also been seen as a significant determinant of preventive behaviour for VBDs^19–22^. Overall, previous studies find that the perception of a high risk of infection or severe disease increases the likelihood of protective behaviours and the number of preventive activities adopted.

Several factors underlie risk perception. Besides knowledge of the mechanisms that affect the occurrence of diseases, risk perception about infections and diseases is jointly determined by personal traits and other factors such as previous life experiences or information received from one’s peers. Thus, risk perception is determined at the individual level and conditioned to both personal characteristics and each individual’s peer group. Specifically, the perception of risk goes beyond individuals and constitutes a social and cultural construct reflecting values, symbols, history, and ideology^23^, which reflect social norms^24^. Social norms have their origins within social groups^25^. There are two types of social groups worth distinguishing: (i) those formed by individuals that actively participate in the enforcement of social norms (e.g., family members, friends, colleagues); (ii) those formed by individuals that conform to similar others based on characteristics such as age, gender, or education, but who are not necessarily related to each other on the social sphere. The latter is named in the Economics literature as the reference group^26,27^.

The SARS-COV-2 pandemic has also drawn attention to the social norm component of preventive behaviour, where social norms can be either enforced through social interactions within groups of individuals sharing a common social space (social group (i)) and sustained by feelings of anxiety, guilt, embarrassment or shame if violated^27,28^, or defined by the most common and expected behaviour from the social group (ii). In the context of SARS-COV-2, it has been highlighted that social norms are constitutive of compliance with preventive measures^29–32^. We argue that social norms also apply to VBDs’ preventive behaviour. However, social norms are likely to influence behaviour differently for SARS-COV-2 and VBDs, possibly due to the different transmission mechanisms of the infection. SARS-CoV-2 transmission occurs directly through social contact among human beings, while a vector mediates VBD transmission. For this reason, SARS-CoV-2-related behaviour is directly influenced by social norms emerging from both types of social groups^33^ while VBD preventive behaviour is likely to be indirectly affected by social groups through their effect on risk perception^34^. As social norms are sustained by comparisons with those forming the reference group, individual risk perception is reported based on, amongst other factors, the perceived reference group’s risk perception.

This work focuses on the relationship between preventive behaviour, risk perception and social norms for selected VBDs, namely Zika virus (ZIKV) disease, malaria, dengue and cutaneous leishmaniasis (CL), in a highly understudied setting such as Guyana, South America. We gather knowledge of social science, public health, epidemiology, including social epidemiology, and entomology to generate innovative policy indications to tackle VBDs. Specifically, we sourced both the theory on social norms and the empirical methods for their identification and measurement from the social science literature and we apply those to VBD infection mechanisms and prevention.

## Methods

### Ethics statement

The study protocol with reference number 265 was reviewed and approved by the Institutional Review Board (IRB) of the Ministry of Public Health of Guyana. The research was conducted in accordance with the principles of the Helsinki Declaration. All adult subjects provided written informed consent prior to participating in the study.

### Data and outcome variables

We undertook a cross-section survey among 845 individuals. We report the sample selection method in the Supplementary Materials C. Of the 845 observations, the analysis was conducted on the 497 collected from private houses; we dropped, therefore, the 348 observations collected from schools, workplaces, health facilities and businesses, as there was no information on socio-economic and demographic characteristics for these observations. Information was collected between August and December 2017 in four regions of Guyana (South America), Region 1 (Barima-Waini), Region 4 (Demerara-Mahaica, the region of the country capital, Georgetown), Region 6 (East Berbice-Corentyne) and Region 8 (Potaro-Siparuni). In the regions of Demerara-Mahaica and East Berbice-Corentyne, information was uniquely collected in the main urban areas, that is, Georgetown and New Amsterdam, respectively. Regions 4 and 6 are coastal; Regions 1 and 8 are in the interior. Data were collected in different regions of the country to capture the heterogenous endemicity of the infections included in this study. In interior regions 1 and 8, where mining is the main economic activity, there is a higher concentration of malaria and CL than in regions 4 and 6^35,36^. While in the coastal regions, arboviruses such as dengue and ZIKV are more prevalent than in regions 1 and 8^37^. Interviews were conducted through a structured questionnaire, which included questions on disease knowledge, risk perception and preventive behaviour for malaria, dengue, ZIKV and CL, together with questions on interviewees' socio-economic characteristics. Additional information on the study can be found in a previously published study that used the same data^19^.

Individuals can rely on non-exclusive active or passive behaviour measures to face risk. Passive behaviour refers to using measures the government provides free of charge as part of the national vector-control programme. On the contrary, active behaviour involves preventive measures that individuals actively acquire. For this study, we considered active behaviour the use of any of the following preventive measures: screened windows, skin repellent, mosquito zapper rackets, beeper mosquito, mosquito coils, bracelets, or simply sitting next to a fire at night. On the other hand, passive behaviour was considered when the reported measures were indoor residual spraying (IRS), fogging or bed nets.

### Empirical strategy

We base our empirical strategy on three postulates: (i) risk perception on different diseases should be estimated jointly given that they depend on common individual traits; (ii) active and passive preventive behaviours are conditioned on risk perception and jointly determined at the individual level, and (iii) social norms play a relevant role when the reference group affects individual risk perception. From an empirical perspective, the first two postulates imply that risk perception and behaviour should be jointly estimated. As for the third one, the reference group for each individual needs to be defined.

According to Manski^38^ and Etilé^27^, identifying the effects of social norms require defining the reference group to which each individual identifies and hinges upon a critical assumption, the exogeneity of the characteristics used to identify the social norm. Within this study, the social norm involves individuals comparing their risk perception to the group’s commonly held norm, which in turn is assumed to influence the individual’s reported behaviour. In other words, being more or less risk averse than the average risk aversion within the reference group affects self-reported risk perception. Hence, we hypothesise that one’s self-reported risk perception is conditioned by a reference group’s inherent risk perception.

According to the literature, those labelled as the reference group share similar characteristics, such as ethnicity, age, gender, or education, but are unrelated to each other in the social sphere. Consequently, this group is intrinsically (more) exogenous than the group formed by those who can actively enforce social norms. In the study, following standard practice, we constructed the risk perception of the reference group as the average reported perception of the reference group once the individual risk perception is removed. Based on the literature, social norms are confirmed by the positive correlation between the group and individual risk perceptions, as introduced by postulate (iii).

Equations (1) to (6) show the details of the simultaneously estimated equations. Alternative specifications were used to accommodate the distribution of each endogenous variable. Equations (1) and (2) were estimated using Tobit, ordered Probit and ordinary least squares (OLS) methods. Equations (3) to (6) were estimated using Probit. To obtain a more straightforward interpretation and convergence of Maximum Likelihood estimates, we applied OLS estimations of risk perception (Eqs. 1, 2) and a Probit model to estimate active/passive behaviour (Eqs. (3) to (6)).

1 $${d}_{r}={S}_{j}^{d}\delta +{r}_{i}+{\epsilon }_{i},$$

2 $${m}_{r}={S}_{j}^{m}\delta +{r}_{i}+{\nu }_{i},$$

3 $${d}_{act}={X}_{i}{^\prime}\gamma +{d}_{r}{\beta }_{1}+{u}_{i},$$

4 $${m}_{act}={X}_{i}{^\prime}\gamma +{m}_{r}{\beta }_{2}+{\mu }_{i},$$

5 $${d}_{pass}={X}_{i}{^\prime}\gamma +{d}_{r}{\beta }_{3}+{\varepsilon }_{i},$$

6 $${m}_{pass}={X}_{i}{^\prime}\gamma +{m}_{r}{\beta }_{4}+{\xi }_{i},$$

where *d* represents dengue, *m* malaria, *S* is the average perception risk for the reference group *j* excluding individual *i*, *X*_*i*_ is the k-vector of explanatory variables, *r*_*i*_ represents precipitation levels during the previous 15 (4) days for malaria (dengue). The last term of each equation represents the residual terms. We considered two dichotomous variables representing an active or passive behaviour against the stated disease risk perception. We excluded from *X*_*i*_ those variables that formed the reference group to avoid collinearity and allow for convergence of estimates. The analysis focuses on malaria and dengue only as both risk perception and behaviour were too low for ZIKV and CL.

When constructing a relevant reference group, we acknowledge that risk perception in health is influenced by factors such as education, income, age, or gender^39^. The size of our dataset constrains the definition of a reference group: while several factors determine the group, the group size needs to be large enough to allow for identification. Therefore, the number of individuals categorised into each combination of conditioning variables limits the number of factors considered when constructing the reference group. Given the characteristics of our population of interest, we tested for several factors that might jointly condition individual risk perception from a social and cultural perspective such as gender, age (quartiles), ethnic group and educational level.

Accordingly, we constructed the reference group, *j*, for each individual, *i*, based on all plausible combinations of these factors that showed enough comparability power, that is, enough sample size within the reference group, resulting in two potential reference groups based on: (1) age and ethnic group, and (2) age and educational level. Our main specification, which considers age and ethnic group as the determinants of the reference group, was chosen based on performance (logarithm of likelihood function) and identification of social norms. Straightforwardly, we chose the best-performing model with a reference group that significantly predicted individual behaviour. The choice of the reference group is aligned with what is consistently depicted in the literature as common determinants of homophily, major sociodemographic dimensions that stratify society, and natural identities that affect behaviour^40–42^.

In addition to the reference group, we included the moving average level of precipitation over a few days preceding the interview, $r$, as an additional covariate that may explain individual risk perception. This additional covariate satisfies the exclusion restriction. That is, we assume that precipitation records could condition risk perception but not active or passive behaviour.

Beyond risk perception, the selection of covariates, $X$, consists of factors that may mediate how individuals react to risk. Such factors are mostly related to economic conditions. Thus, in addition to considering the floor and wall quality of dwellings, we included a measure of deprivation based on a list of 30 reported owned assets. Specifically, we used a Rausch model (a joint maximum likelihood estimator for Rausch Item Response Theory models of dichotomous items) to compute “wealth” scores for each individual^43^. Also, we included demographic characteristics, such as gender, ethnic group, marital status, education, and having relatives living abroad (the diaspora outside Guyana has been a major phenomenon for many years^44^). Finally, we controlled for region-fixed effects. For estimation purposes, when a reference group included any of these characteristics, we excluded them from the list of covariates.

### Robustness checks

We performed a secondary analysis and sensitivity analyses to test the robustness of our results to some of the definitions adopted in our main specification. As a secondary analysis, we estimated the same model by altering the reference group: age and educational level. In this case, we excluded education from the list of covariates, X, in regressions (3)–(6) but included the deprivation measure. The deprivation index was not considered in our main specification because of our sample’s high correlation between wealth and educational levels. As a sensitivity analysis, we first consider active/passive behaviour as an index of reported behaviours instead of a dichotomous variable. That is, we constructed a count of all reported active/passive behaviours for each disease. In this case, all six regressions were estimated by ordered probit.

Second, we tested the sensitivity of our results to the exclusion of bednets as a (passive) measure. The reason for such exclusion is bednets can also be directly bought by individuals as a substitute or in addition to those distributed by (1) the Ministry of Health during mass campaigns, at the health facilities or on health outreaches; (2) camp managers to miners on the workplace. In our main specification, the reason why we considered bed nets as passive behaviour is twofold: (i) liquidity constraints have been seen as major barriers towards bednet purchase and, in the absence of subsidies, the ownership rate is likely to be low^45^; (ii) a large effort is put in place by the government of Guyana to distribute highly effective long-lasting impregnated nets (LLINs) to fight, primarily, malaria. A large governmental distribution campaign was conducted in 2018, 1 year after our survey. Despite that, anticipatory effects may have shaped the population's behaviour towards not actively acquiring bed nets as the government conducted sensitisation campaigns before the actual bednet distribution.

## Results

**Figure 1:**
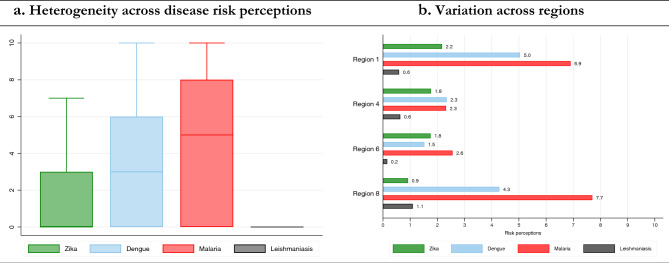
Variation in risk perception.

The final analysis was performed on 488 individuals with complete cases. Risk perception across the selected diseases showed different patterns. Average/median risk scores on a 0–10 scale were the following: ZIKV (1.68/0), dengue (3.39/3), malaria (4.94/5) and CL (0.63/0). The overall unweighted average risk perception turned out to be 2.66 (median = 2.75). Figure 1a displays boxplots of the risk perception distribution for each disease. We observe that CL, mostly unknown across regions, was not perceived as a risk. In contrast, individuals were more risk-averse against malaria, showing the highest heterogeneity across the four diseases (largest interquartile range (IQR)). Dengue and ZIKV were next in risk perception descending order. To correct for misreporting, we set risk perception as 0 when the individual had no knowledge about the disease. The underlying idea is that, in line with the KAP model theory, individuals cannot hold attitudes toward something they are unaware of. As could be expected, the magnitude of pairwise correlations between knowledge and risk are remarkable: ZIKV (0.68), dengue (0.71), malaria (0.50) and CL (0.61). Figure 1b shows that individuals in coastal areas (regions 4 and 6) have higher relative risk perception for ZIKV and Dengue fever compared to hinterland regions, accordingly to the incidence of these diseases in those regions.

**Figure 2:**
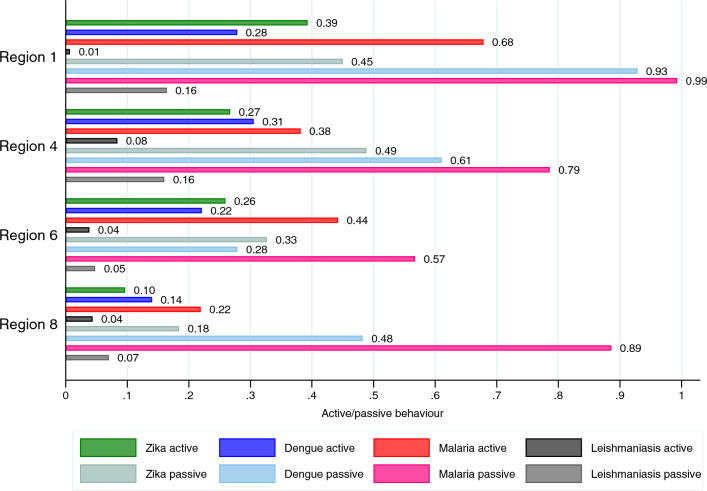
Variation across regions in active/passive measures.

As expected, active measures were, on average, used by a significantly lower proportion of individuals compared to passive ones: ZIKV (26.2% vs 37.2%, Chi^2^ = 97.34, p < 0.001), dengue (24.1% vs 60.1%%, Chi^2^ = 63.97, p < 0.001), malaria (44.2% vs 82.2%%, Chi^2^ = 42.66, p < 0.001) and CL (4.3% vs 11.7%%, Chi^2^ = 132.38, p < 0.001). Figure 2 displays heterogeneity across regions. Notably, Region 8 showed the lowest active average behaviour compared to regions where the prevalence of malaria is lower. ZIKV and CL were excluded from the analysis because the observed risk perception for both diseases does not show enough statistical variation.

Supplementary Figures A1–A3 (Supplementary Materials A) show risk perception according to education, ethnicity and age. While dengue risk perception is not correlated with age (p > 0.05), it is significantly correlated with ethnicity and education (Chi^2^ = 24.77, p = 0.006 and Chi^2^ = 84.17, p < 0.001, respectively). In the case of malaria, risk perception is significantly correlated with the age group (Chi^2^ = 66.56, p < 0.001), education (Chi^2^ = 31.63, p < 0.001) and ethnicity (Chi^2^ = 119.08, p < 0.001), with Amerindians having the higher risk perception in comparison the other ethnic groups.

Supplementary Figures A4–A6 (Supplementary Materials A) show similar behaviour coverages, active and passive, for dengue disease across age groups. We find significant correlations between dengue active behaviour and education (Chi^2^ = 10.07, p = 0.002), and dengue passive behaviour and ethnicity (Chi^2^ = 24.66, p < 0.001). For malaria, we observe differing active and passive behaviour across age groups (Chi^2^ = 15.60, p = 0.001 and Chi^2^ = 12.91, p = 0.005, respectively) and ethnicity (Chi^2^ = 13.35, p = 0.004 and Chi^2^ = 40.52, p < 0.001, respectively). The highest coverage (96%) is recorded for malaria passive behaviour among the Amerindian ethnic group. In addition, the two education groups differ in the reported active behaviour (Chi^2^ = 17.97, p < 0.001).

Table 1 shows the list of covariates that may condition risk perception and preventive health behaviours included in our empirical analysis. The average (median) age of our sample is approximately 42 (41) years, with a significantly higher prevalence of female individuals (76%). Our sample is evenly spread across the four regions and across four ethnicities: African (26%), Amerindian (24%), East Indian (20%), and mixed (30%). The deprivation index categorised individuals into five wealth groups: low (22%), low-middle (25%), middle (18%), upper-middle (22%), and high (14%). Floors were reported of higher average quality than walls, with considerably lower reporting of rotten floors (3%) compared to bad quality walls (36%). In our sample, 35% reported being married, 33% in a common law partnership and 24% single. A marginal number of individuals reported being either separated/divorced (4%) or widowed (4%). In terms of education, the highest proportion of individuals reported having either secondary or tertiary level (69%). Sixty-five per cent reported having at least one household member living abroad.

**Table 1:** Summary statistics of covariates.

Variables	Mean/frequency (std. dev.)
Age	41.84 (15.83)
Female	0.76 (0.43)
Ethnic group	
African	0.26 (0.44)
Amerindian	0.24 (0.43)
East Indian	0.20 (0.40)
Mixed	0.30 (0.46)
Region	
Region 1	0.29 (0.45)
Region 4	0.27 (0.44)
Region 6	0.21 (0.41)
Region 8	0.23 (0.42)
Deprivation index	
Low income	0.22 (0.41)
Low-middle income	0.25 (0.43)
Middle income	0.18 (0.38)
Upper middle income	0.22 (0.41)
Higher income	0.14 (0.35)
Floor quality	
Bad floor quality	0.03 (0.18)
Average floor quality	0.51 (0.50)
Good floor quality	0.46 (0.50)
Walls quality	
Bad walls quality	0.36 (0.48)
Average walls quality	0.37 (0.48)
Good walls quality	0.27 (0.45)
Abroad	
No relatives abroad	0.35 (0.48)
Relatives abroad	0.65 (0.48)
Marital status	
Common law	0.33 (0.47)
Married	0.35 (0.48)
Separated/divorced	0.04 (0.20)
Single	0.24 (0.42)
Widow/widower	0.04 (0.21)
Educational level	
Primary or less	0.31 (0.46)
Secondary/tertiary	0.69 (0.46)

**Figure 3:**
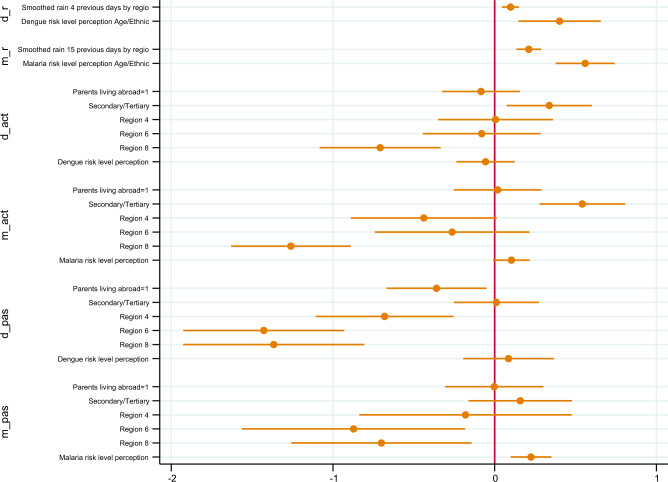
Estimation results: simultaneous marginal effects (social norms: age and ethnic group).

Figure 3 shows the marginal effects of the six-equations model. Both precipitation and the risk perception of the reference group were relevant and positively associated with the individual’s risk perception (Eqs. 1, 2). Thus, the more abundant the rain on the days prior to the interview, the higher the risk perception. The greater the risk perception of the reference group, the higher the individual risk perception.

After considering a simultaneous joint decision by individuals, risk perception was positively statistically associated with passive measures adopted for malaria (p < 0.001). Hence, the higher the risk perception for malaria, the greater the likelihood of accepting passive measures against the infection. Per each additional point increase in the 10-point Likert scale, the likelihood of acceptance of passive malaria preventive measures increases by 4.48% (95% CI 2.10–6.86%). Risk perception does not predict behaviour in the case of dengue and only weakly predicts malaria active behaviour (p = 0.074).

Regarding other predictors of active/passive behaviour, higher education levels are associated with active preventive behaviours against infections but not with passive ones. There are regional differences compared to the hinterland base category (Region 1). Specifically, individuals based in Region 8 showed lower probabilities of carrying out both active and passive measures and individuals from coastal areas (Regions 4 and 6) were less willing to tackle dengue passively. People in Region 6 were also less prone to uptake passive protective measures against malaria. In coastal areas, no statistical differences were found for active behaviour.

**Figure 4:**
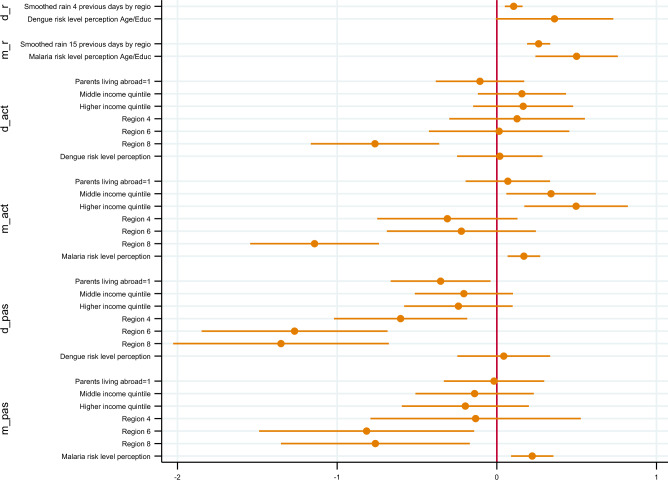
Estimation results: simultaneous marginal effects (social norms: age and educational levels).

When estimating the model with age and education as an alternative definition of the reference group, results remain consistent in describing the relationship between malaria risk perception and passive preventive behaviour (Fig. 4). Nevertheless, malaria risk perception becomes statistically significant both for passive and active behaviour (p < 0.001). Increased malaria risk perception is associated with an increased (willingness to) uptake of both active and passive behaviours against the disease by 5.50% (95% CI 2.67–8.33%) and 4.42% (95% CI 1.96–6.88%), respectively, per unitary increase in the risk perception scale. Moreover, the risk perception of the reference group based on age and education ceases to explain dengue individual risk perception, pointing, thus, to the absence of a social norm. Last, results show that higher income levels were positively correlated to active behaviour exclusively for malaria, in contrast to education which influenced active behaviour irrespective of the disease.

Supplementary Figures B1 and B2 show that results are also consistent when considering active/passive behaviour as an index: risk perception predicts the willingness to use passive measures against malaria. However, when we exclude bednets from the analysis, leaving IRS and fogging as the only passive behaviour, results vary (Supplementary Figs. B3, B4, in the Supplementary Material B). Specifically, irrespective of the definition of the reference group, passive behaviour against dengue is explained by risk perception (p < 0.001), while risk perception ceases to be significant in the malaria passive behaviour equation.

## Discussion

Understanding the extent of the social determinants of preventive behaviour against vector-borne diseases (VBDs) is key to drawing effective health policies. When social norms are proven to influence individual behaviour, tailored interventions can be targeted to identified social groups, potentially multiplying the effects of policy interventions^46^. This is particularly important in areas of low transmission where disease elimination is within reach. Still, the risk perception is likely to be low, and the path towards the last mile entails the need for nudging preventive behaviours through, for example, the spread of information on the risk of outbreaks and resurgences^47,48^.

This study analysed the relationship between individual risk perception and preventive behaviour towards selected VBDs. In addition, we investigated the social dimension of individual preventive behaviour by testing, within identified reference groups, the existence of social norms influencing individual risk perception and, consequently, individual preventive behaviour. Due to the very nature of vector-mediated transmission mechanisms of VBDs, we considered social norms that emerge from the reference group, that is a group of individuals that share similar traits but are not necessarily linked in the social sphere. We hypothesised that social norms exist through the mediation of risk perception.

Descriptively, we found that both risk perception and behaviour, particularly passive behaviour, were higher for malaria than for any other VBD. The infection that ranked second was dengue. Risk perception and passive behaviour were the highest in regions 1 and 8 for malaria and dengue. In contrast, Region 8 had the lowest reporting of active behaviour, probably due to regional wealth disadvantages (see Supplementary A, Table A1)^49–51^. The lowest levels of risk perception and corresponding behaviour were observed for CL. This can be attributed to the high prevalence of individuals with no knowledge about CL in our sample, resulting in a sample average risk perception for CL at marginally negligible levels (see Fig. 1). Disease awareness may also be the driver of the results on the association between active behaviour and socioeconomic factors (specifically, education and income). Malaria, being the most widely known disease in our sample, shows a positive correlation between active behaviour and income. In contrast, dengue, with equally lower knowledge levels across income groups, lacks a significant correlation between income and active behaviour. In the case of education, disease awareness is concentrated in the higher education groups, which would favour education being associated with active behaviour for both diseases. Knowledge was not included in our model due to a dimensionality issue paired with a collinearity issue arising when knowledge is used to predict both risk perception and behaviour.

These figures reflect the available information on the epidemiology of the infections and the dissemination of information to the population. As reported in a previous study, malaria was on the rise between 2016 and 2017, with the number of yearly cases being around 13,000^19^ and there is also evidence of increasing incidence at the borders between Brazil, Guyana and Venezuela^52^. The number of cases detected is far lower for dengue and ZIKV and even lower for CL. In Guyana, Vector Control Services and the Ministry of Public Health have made enormous progress with regard to the dissemination of information on VBDs among the population at risk. However, the existing surveillance system is fed from a manual data collection process, and due to limitations in the timeliness of reporting, there are lags in receiving data from remote areas^53^. Therefore, possible outbreak scenarios are often flagged through informal reports from community members rather than the surveillance system. These reports are often captured in the media—mostly in the form of unspecified increases in cases or deaths. More generally, the reporting of VBDs in mainstream media is event-based and features in cases of an outbreak, to inform about public campaigns or initiatives, or should there be a commemoration of an important national or international effort or period such as Mosquito Awareness Week in May. In the recent past, for arboviral diseases, there were few media reports on ZIKV, Chikungunya and dengue. Malaria often features the case burden rather than the number of deaths. Minimal attention is placed on CL, with outbreaks primarily concentrated in specific areas, especially those with a high transmission rate due to mining activities. Given that our study was not undertaken during or in time proximity to any disease outbreaks, we do not have reasons to think that the release of information was skewed towards any of the diseases included in our analysis. That is, we do not expect the replies received during data collection to have been particularly affected by health communication.

We found that the reference group influences individual risk perception both for malaria and dengue. In the case of malaria, this was apparent for the two definitions of reference group: (i) age and ethnic group, and (ii) age and education. The results show that risk perception significantly and positively affects malaria preventive behaviour, both passive and active, when the reference group is based on age and education; and passive malaria behaviour when the reference group was based on age and ethnic group. However, the association with passive behaviour disappeared when bed nets were withdrawn from the analysis. That is, risk perception only predicted the uptake of passive malaria behaviour when it included the acceptance and usage of bednets. This highlights the perceived high effectiveness of bednets as a tool against malaria. Similarly, the exclusion of bednets as a form of passive behaviour points out the importance of IRS/fogging as a prevention tool against dengue. As results show, risk perception is a positive and significant determinant of dengue passive behaviour when bednets are excluded from the analysis, and only fogging is considered. The sensitivity analysis results showcase that interviewees correctly disentangle which preventive tools are more efficacious for each disease. In fact, while there is some evidence pointing to the efficacy of bednets in reducing the *Aedes aegypti* mosquito’s population, which is responsible for dengue transmission and bite during daylight time^54^, bednets remain a main entomological strategy for impeding contact between humans and the mosquito responsible of malaria transmission—predominantly at night-time, the *Anopheles*.

Less clear-cut results were found for the remaining VBDs. ZIKV and CL were excluded from the analysis as both risk perception and behaviour were too low. For dengue, the role of social norms in determining risk perception was weaker: the relationship between the reference group and the individual risk perception was dependent on the definition of the reference group. More specifically, we only observe the presence of social norms when the reference group is based on age and ethnicity. Thus, when considering other definitions of the reference group, individual risk perception seems to be explained by factors other than the risk perception of the reference group. Additionally, the role of risk perception in influencing dengue preventive behaviour depends on the definition of passive behaviour: risk perception is only a relevant factor in the uptake of passive behaviours such as fogging and IRS.

In the previous study that used the same data^19^, the aim was to understand the contribution of disease knowledge and risk perception on passive behaviour, while no social norm dimension was included in the analysis. Analytically, a different model was estimated from the current one and all four VBDs were included in the analysis. Despite the conceptual and empirical strategy differences, individual risk perception was also found to positively and significantly contribute to passive behaviour, for all four diseases.

An important finding is that despite not engaging in active behaviours as a reaction to higher levels of (perceived) risk of infection, individuals are willing to accept or use passive measures to protect themselves. This finding should favour the government’s provision of protective tools against both dengue and malaria^55^. Furthermore, given the relevance of social norms in explaining malaria risk perception, interventions such as health communication and promotion activities tailored to groups based on key demographic and social characteristics (i.e., age, ethnicity and education) should produce a replicative effect and reinforce malaria preventive behaviours. For example, information and awareness campaigns on the actual current and future infection, morbidity and mortality risk should accommodate individual characteristics in order to reinforce the uptake of distributed protective measures against malaria. On the one hand, there is evidence that crafting messages based on the beliefs and motivations of individuals is highly effective^56–58^. On the other hand, it has been proven that the way people interact, interpret and participate in health promotion interventions varies depending on the intersections of ethnicity and demographic variables such as age and gender^59,60^.

In an intent to define tailored communication strategies in the context of our study, we draw upon key insights from the literature on health communication. The strategy proposed can be linked to the work of Hawkins et al.^61^, who reduce the individualisation of health communication to an appropriate segmentation of the population and the customisation of the source, message, and channel of communication to effectively reach each segment. The results of our study suggest a segmentation of the target audience based on demographic characteristics such as age, ethnicity, and education. One common strategy in the customisation process is the personalisation of messages through contextualisation. By framing our messages in contexts that resonate with the personal background of the recipients, we can significantly enhance their attention, interest and motivation to actively and thoughtfully process information^58,61,62^. For instance, studies in public health have already shown that age-specific messaging can improve health behaviour outcomes among different age groups^63^. Moreover, incorporating the ethnic and educational context of each recipient should involve health communication tailoring ensuring cultural sensitivity, linguistic appropriateness and literacy representation within the target audience.

The findings from this study could be extrapolated to similar settings, e.g. Suriname, with comparable disease epidemiology, common socio-economic conditions (e.g. an important economic activity, such as mining, highly linked to malaria infection), and the movement of people between countries, with the associated sharing of knowledge and experiences. All in all, evidence should be generated in Guyana and similar settings on the impact on preventive behaviour for VBDs of interventions aimed to correct risk perception tailored to groups at the intersection of certain socio-economic and demographic characteristics.

### Study limitations

This study had some limitations that need to be highlighted. Firstly, the sample size limits the reference groups considered for analysis. Due to a dimensionality issue, the number of factors considered at once, when constructing the reference group, is restricted to two. Larger sample sizes would grant the opportunity to better analyse intersectionality in reference groups. Secondly, the study is based on data collected at one point in time in one country, Guyana. Despite we suggest that results could be extrapolated to similar settings, they cannot be generalised beyond similar settings. Studies including countries or areas with heterogenous epidemiological, cultural and socio-economic conditions would increase the external validity of findings. Third, we use retrospective data from pre-pandemic times. The COVID-19 pandemic may have mediated the relationship between social norms, risk perception and preventive behaviour.

### Supplementary Information

Supplementary Information.

## Data Availability

The datasets generated during and/or analysed during the current study are available from the corresponding author upon reasonable request.
