# Supplementary material for: The contribution of risk perception and social norms to reported preventive behaviour against selected vector-borne diseases in Guyana
Source: Sci Rep. 2023 Oct 6;13:16866. doi: 10.1038/s41598-023-43991-1 (PMC10558444; doi:10.1038/s41598-023-43991-1)
Supplement: Supplementary file 1 — Supplementary Information. [file 41598_2023_43991_MOESM1_ESM.pdf]

Supplementary materials to the article:

The contribution of risk perception and social norms to reported preventive behaviour against selected vector-borne diseases in Guyana

Iris Lopes-Rafegas[1*], Horace Cox[2], Toni Mora[3], Elisa Sicuri[1,4*]

1. ISGlobal, Hospital Clínic Universitat de Barcelona, Barcelona, Spain.

2. Vector Control Services, Ministry of Health, Guyana

3. Research Institute for Evaluation and Public Policies (IRAPP), Universitat Internacional de Catalunya (UIC), Barcelona, Spain.

4. LSE Health, London School of Economics and Political Science, London, UK

* Corresponding authors: Iris Lopes-Rafegas (iris.lopes@isglobal.org); Elisa Sicuri (elisa.sicuri@isglobal.org).

[A] Descriptive information

Figure A1. Variation across education groups in risk perception

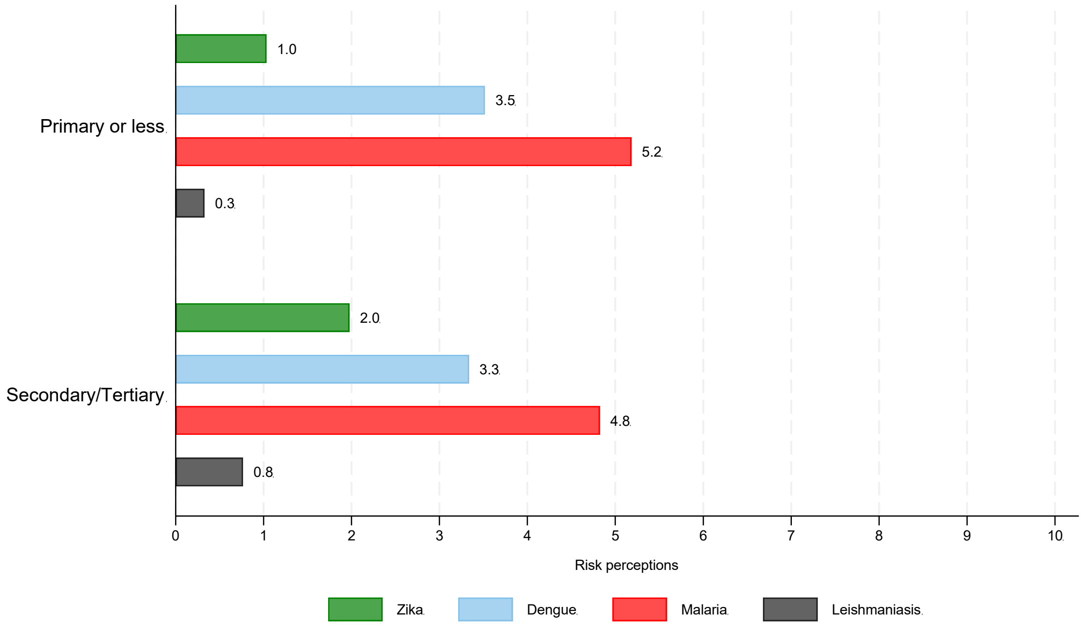

Figure A2. Variation across ethnic groups in risk perception

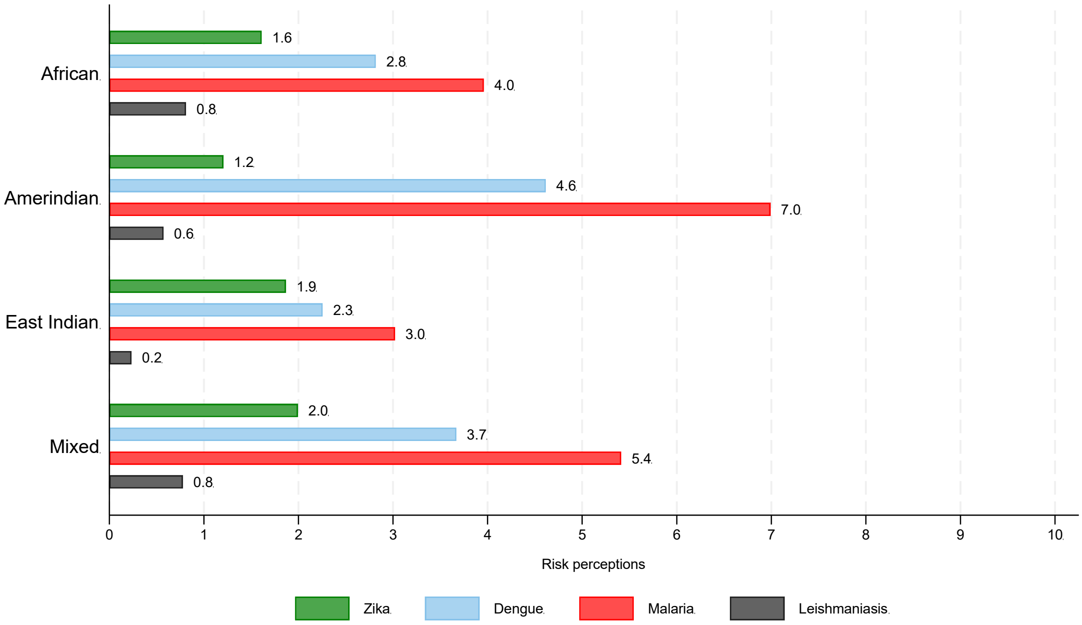

Figure A3. Variation across age groups in risk perception

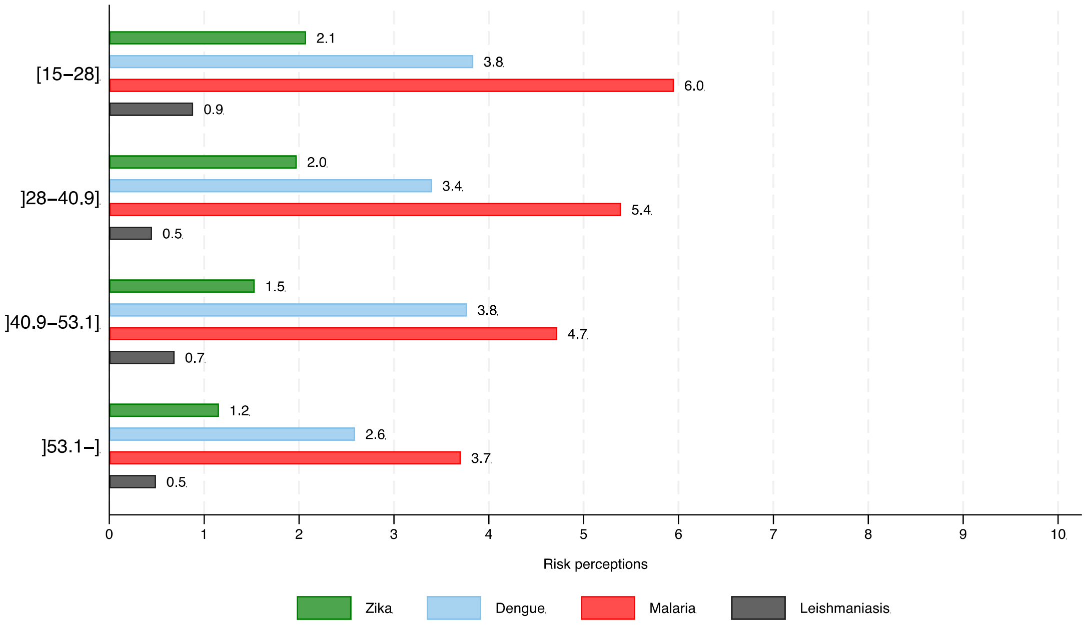

Figure A4. Variation across education groups in active/passive measures

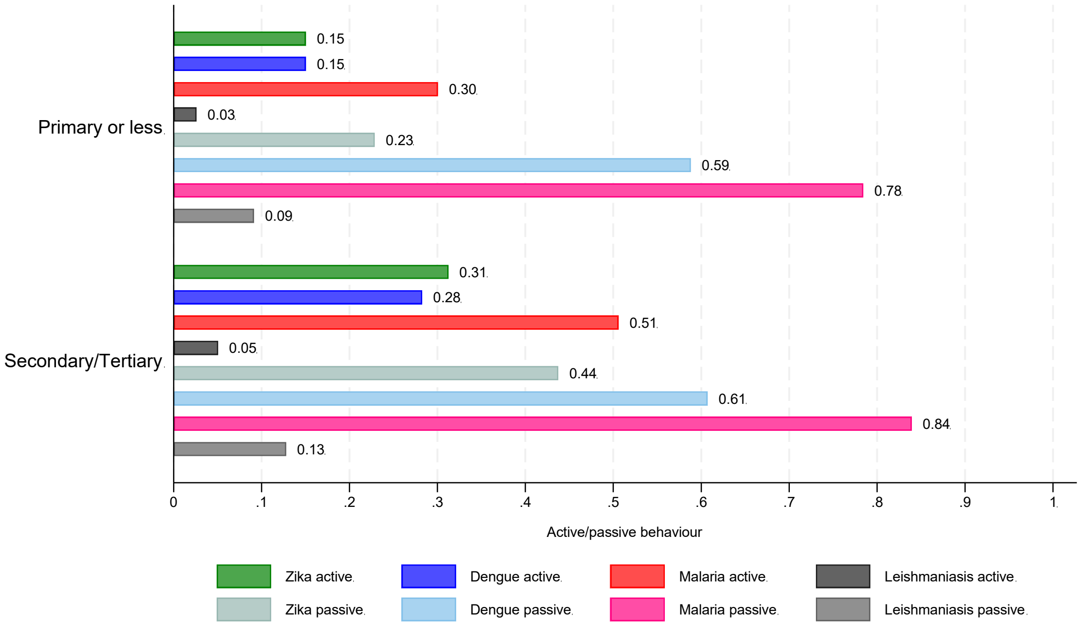

Figure A5. Variation across ethnic groups in active/passive measures.

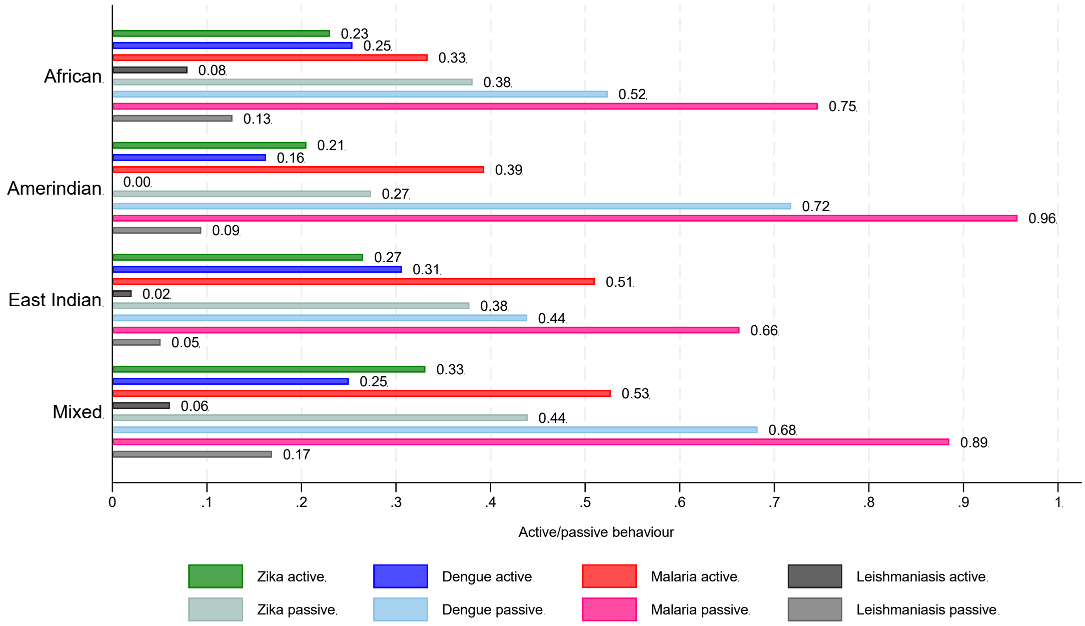

Figure A6. Variation across age groups in active/passive measures.

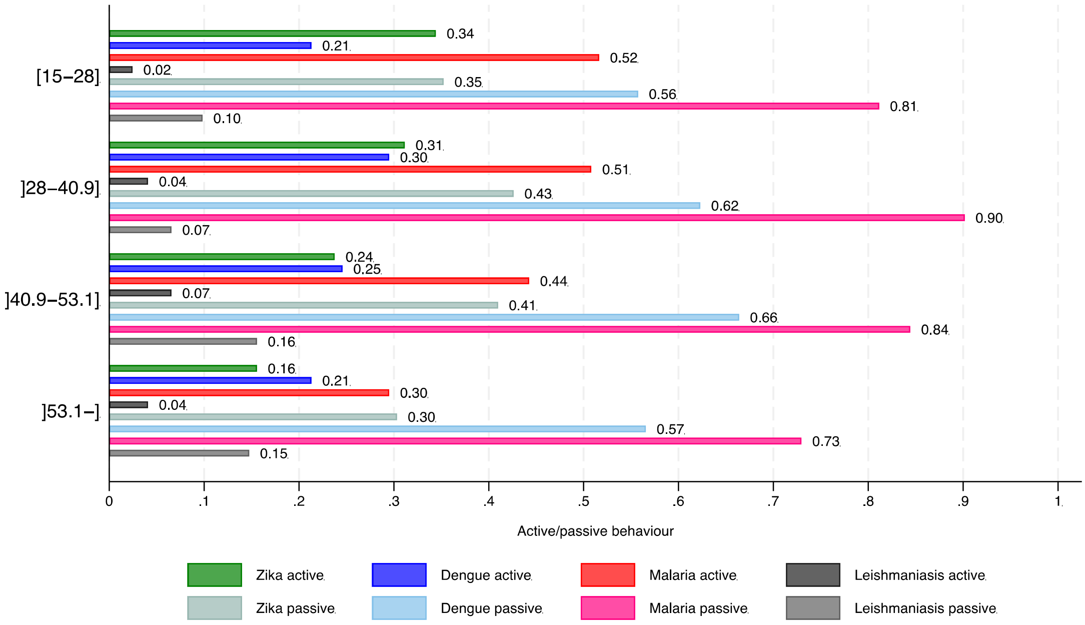

Table A1. Distribution of deprivation index by region

	Deprivation index				
	**N observations**	**Mean**	**Standard Deviation**	**Minimum**	**Maximum**
**Region 1**	140	1.643	0.778	1	3
**Region 4**	131	2.114	0.771	1	3
**Region 6**	104	2.442	0.722	1	3
**Region 8**	114	1.465	0.567	1	3

[B] Sensitivity Analysis

Figure B1: Estimation results: simultaneous marginal effects (social norms: age and ethnic group). Index as the dependent variable.

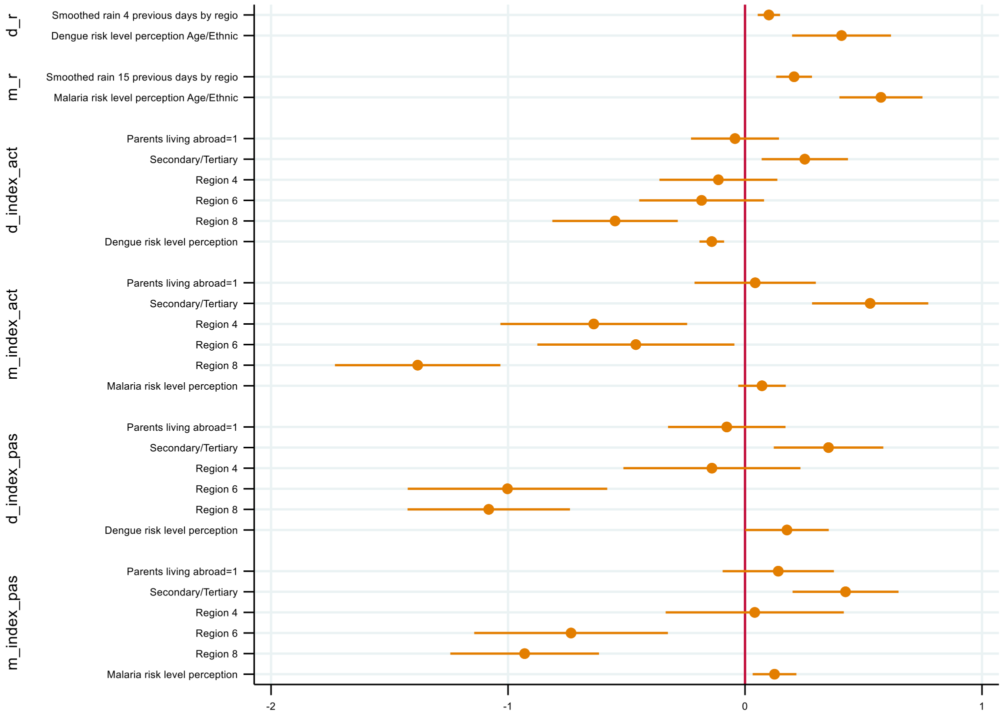

Figure B2: Estimation results: simultaneous marginal effects (social norms: age and education group). Index as the dependent variable.

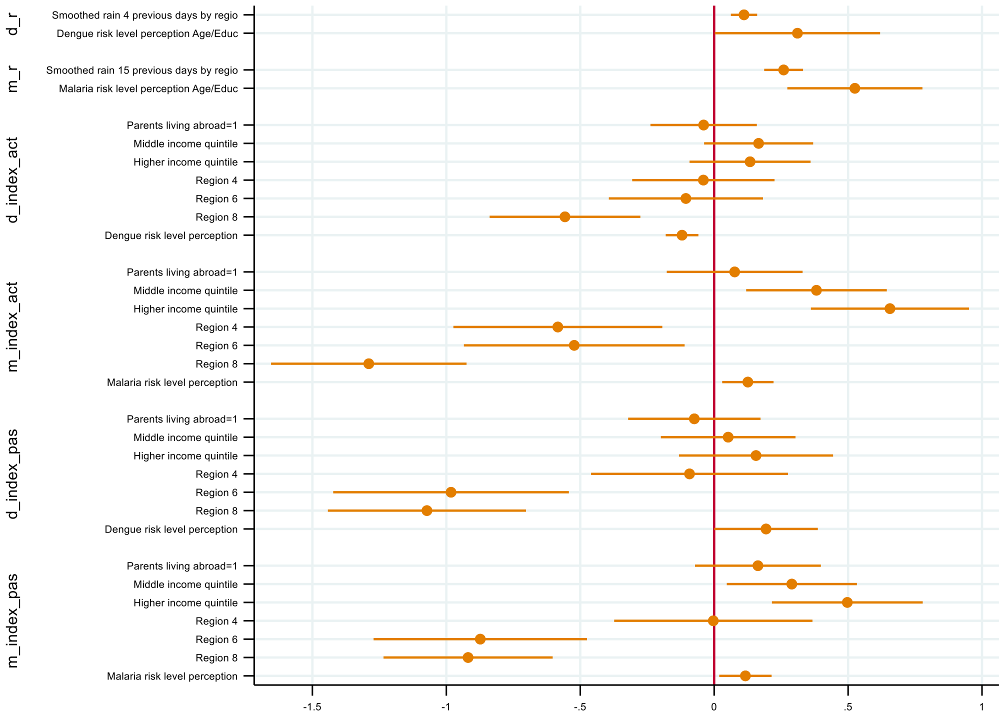

Figure B3: Estimation results: simultaneous marginal effects (social norms: age and ethnic group). The passive behaviour definition excludes bednets.

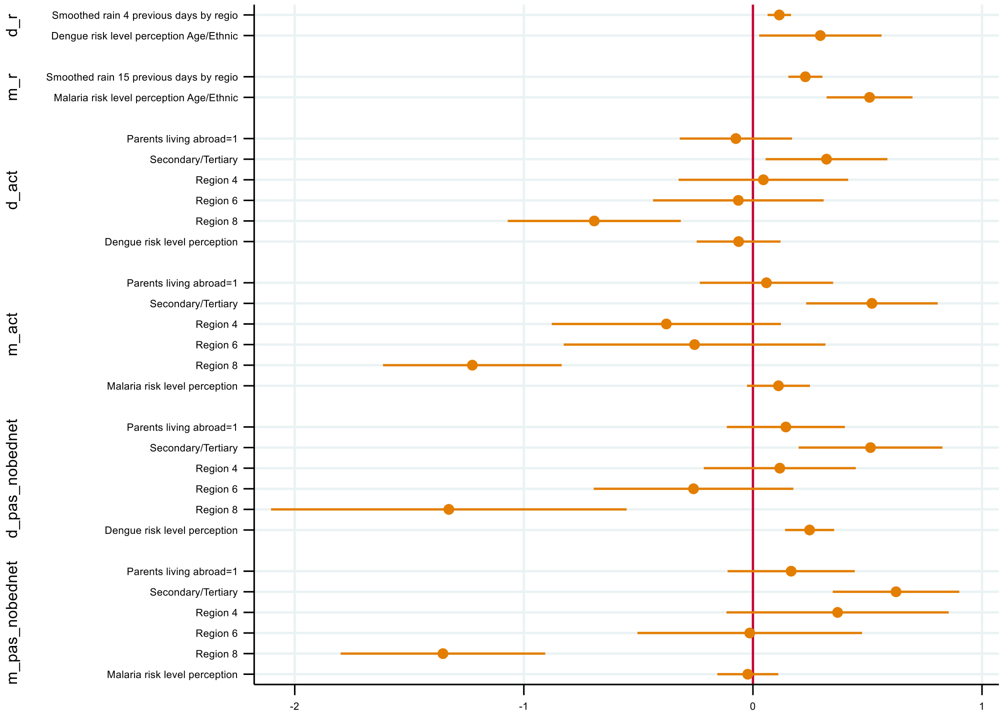

Figure B4: Estimation results: simultaneous marginal effects (social norms: age and education group). The passive behaviour definition excludes bednets.

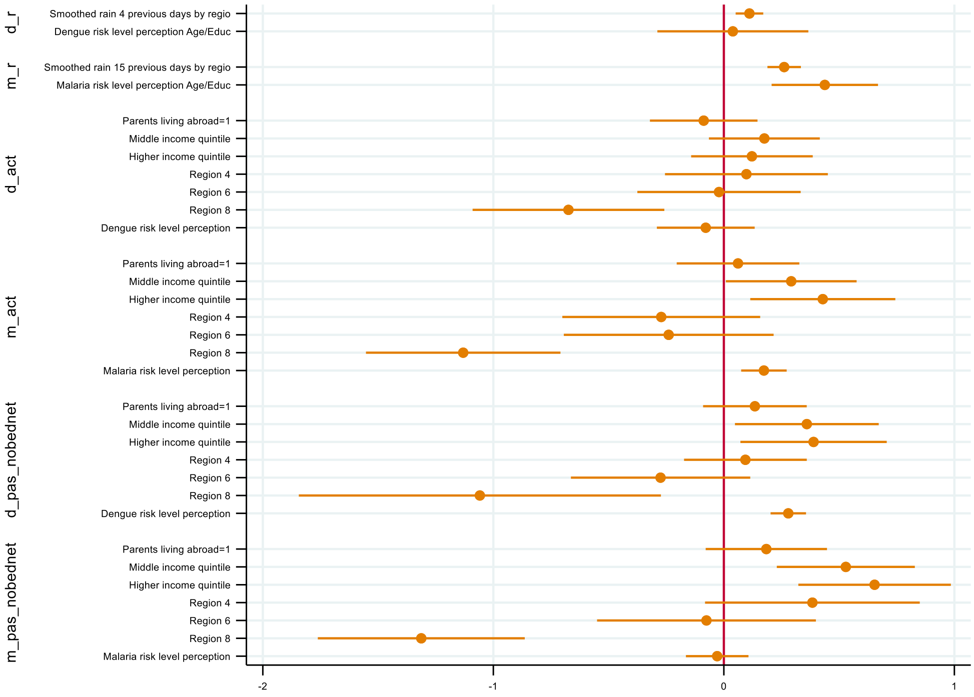

[C] Sample Selection Method

The sample size was strongly determined by the availability of resources. Therefore, the sampling approach was exploratory. Within this context, the aim was representing both coastal and hinterland regions, which explain a substantial within-country variability across a number of relevant dimensions for this study. Given the resource constraint, two coastal and two hinterland regions were selected. Coastal regions include regions 4 and 6, the former includes the capital city Georgetown which concentrates 30% of the national population while the latter includes the second largest city, New Amsterdam. The selected interior regions were regions 1 and 8, which are geographically vast but with a very low population density. The survey was conducted in the vicinity of its two main towns: Mabaruma and Mahdia, respectively.

Overall, 800 questionnaires were collected, which were equally divided across the 4 regions (i.e. a sample size per region of 200). Information on the number of inhabitants in each region, divided into lower administrative units (referred to as villages), was obtained from the Bureau of Statistics of Guyana. The most recent population data available were from the 2012 national census. The Ministry of Public Health selected a subset of villages based on their proximity to the starting point and accessibility by walking distance or public transport. More precisely, 49 villages out of 198 villages were 'preselected' for Region 4 and 22 out of 190 villages for Region 6. Among these villages, 15 villages in regions 4 and 6 were randomly chosen applying sampling proportional to size. In each of the selected villages, the number of questionnaires was also assigned based on population size (Table C1). We assigned 210 questionnaires to account for potential attrition[1]. A starting point was chosen by data collectors within each village who then applied the "spinning bottle" rule. In the interior of the country, due to the small size, significant distance between villages, and limited connectivity, the research was concentrated around the primary centres of Mabaruma in Region 1 and Mahdia in Region 8. Each of these villages had a total population of approximately 2,000 inhabitants. Unlike in other areas, a proportional selection based on size was not utilized for these two towns. Instead, data collectors initiated the selection process from the health centre and proceeded by applying the "spinning bottle" rule.

[1] The study proceeded with 2 additional data collection rounds among the same cohort of individuals.

Table C1: Selection proportional to size (regions 4 and 6)

Names (or abbreviations) of your primary sampling units	Estimated size of sampling units	Probability of inclusion	Number of quesitonnaires
**Region 4**			
Cummings Lodge	7246	1	21
Kitty	6789	1	20
Turkeyen	6599	0,978557447	19
Campbellville	5031	0,74604069	18
Pattensen	5013	0,743371493	17
West Ruimveldt	4206	0,623702473	16
Albouystown	3838	0,569132214	15
Sophia	3687	0,546740613	14
Liliandaal	3100	0,459695118	13
Werk En Rust	2760	0,409276944	12
Albertown	2357	0,349516579	11
East La Penitence	1984	0,294204876	10
Ogle	1391	0,206269648	9
Prashad Nagar	1013	0,150216502	8
Lamaha Gardens	638	0,094608221	7
**Region 6**			
Cumberland	3875	1	21
Mount Sinai	3861	1	20
Canefield	3268	1	19
Rose Hall	3067	1	18
Stanleytown	3049	1	17
Glasgow	1868	0,94448377	16
Adelphi	1303	0,658812822	15
Little Bleyendaal	1214	0,613813328	14
Sheet Anchor	1114	0,563252098	13
Edinburg	1069	0,540499545	12
Reliance	921	0,465668925	11
Ordnance Fort Canje	883	0,446455658	10
Overwinning	873	0,441399535	9
Vrymans Erven	802	0,405501062	8
Palmyra or No. 4	540	0,27303064	7

NB: 210 questionnaires were aimed in each region to account for dropouts in the following rounds of data collection

[D] Questionnaire - Vector Control Services

Vector Control Services - Repuls Project

1. GPS coordinates

latitud (x.y °)

longitud (x.y °)

altitud (m)

precisión (m)

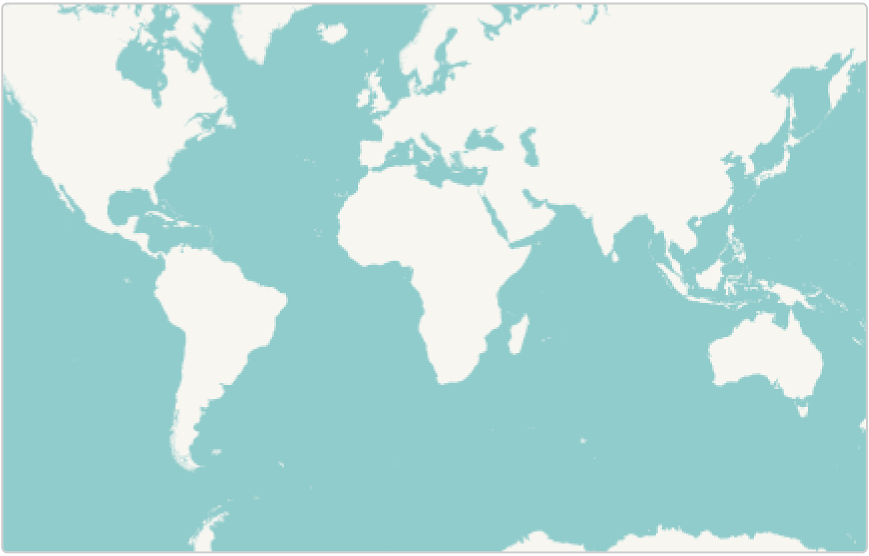

2. a. Region

- Region 1
- Region 4
- Region 6
- Region 8

2. b. Questionnaire serial number

3. Interviewee ID

Enter a 6-digit number after 'abc'

abc

4. Date of interview

Today's date

yyyy-mm-dd

5. Place of interview

- ◯ Private house
- ◯ Workplace
- ◯ Restaurant
- ◯ Hospital/Health centre
- ◯ School

6. Position of interviewee within the place in Question 5

- ◯ Husband
- ◯ Wife
- ◯ Workplace owner
- ◯ Workplace worker
- ◯ Hospital director
- ◯ Hospital worker
- ◯ School director
- ◯ School teacher

7. Nationality

- ◯ Guyanese
- ◯ Brazilian
- ◯ Venezuelan
- ◯ Cuban
- ◯ Other

7. b. Other nationality

____

8. Town / Village name

____

9. Sex

- ◯ Male
- ◯ Female

10. Date of birth

yyyy-mm-dd
____

11. What ethnic group do you belong to?

- ◯ Amerindian
- ◯ European
- ◯ African
- ◯ East Indian
- ◯ Portuguese
- ◯ Chinese
- ◯ Mixed

12. What is your martial status?

- ◯ Common law
- ◯ Married
- ◯ Separated/Divorced
- ◯ Widow/Widower
- ◯ Single

13. What is your highest level of education?

- ◯ Never been to school
- ◯ Primary
- ◯ Secondary
- ◯ Undergraduate studies
- ◯ Postgraduate studies

14. What is your occupation?

- ◯ Farmer
- ◯ Miner
- ◯ Fisherman
- ◯ Office employer
- ◯ Shop trade
- ◯ Other

14. b. Other occupation

____

15. What is your main source of drinking water?

- Piped water - Piped into dwellinger
- Piped water - Piped to yard/plot
- Piped water - Public tap/standpipe
- Piped water - Tube well or borehole
- Rainwater
- Tanker truck
- Cart with small tank
- Surface water (river/dam/lake/pond/stream/canal/irrigation channel)
- Bottled water
- Dug well
- Protected well
- Unprotected well
- Water from spring - Protected spring
- Water from spring - Unprotected spring

16. What kind of toilet facility do you use?

- No facility/bush/field
- Flush to piped sewer system
- Flush to septic tank
- Flush to pit (latrine)
- Flush to somewhere else
- Flush, don't know where
- Ventinlated improved pit latrine
- Pit latrine with slab
- Open pit
- Composting toilet
- Bucket toilet
- Hanging toilet / hanging latrine

17. Do you have:

- [ ] Electricity
- [ ] A radio
- [ ] A cell phone
- [ ] A land-line phone
- [ ] A refrigerator
- [ ] A clock
- [ ] A black/white television
- [ ] A color television
- [ ] A freezer
- [ ] An electric generator
- [ ] A fan
- [ ] An air-conditioner
- [ ] A fan
- [ ] An air-conditioner
- [ ] Washing machine
- [ ] Computer
- [ ] Digital photo-camera
- [ ] Non-digital photo-camera
- [ ] A VHS player
- [ ] A DVD player
- [ ] A bed
- [ ] A vanity
- [ ] A wall divider
- [ ] A watch
- [ ] A bicycle
- [ ] A motorbicycle
- [ ] A motorbicycle or motor scooter
- [ ] An animal-drawn cart
- [ ] A car, truck or mini-van
- [ ] A boat with a motor
- [ ] A boat without a motor

18. What type of fuel do you mainly use for cooking?

- ☐ Electricity
- ☐ LGP
- ☐ Natural gas
- ☐ Biogas
- ☐ Kerosene
- ☐ Coal, Lignite
- ☐ Charcoal
- ☐ Wood
- ☐ Straw/Shrubs/Grass
- ☐ Agricultrual crop
- ☐ Animal dung
- ☐ No food cooked in household

19. Does any member of this household own any agricultural land?

- ○ Yes
- ○ No

20. Which of the following animals does this household own?

- ☐ Milk cows or bulls
- ☐ Horses, donkeys or mules
- ☐ Goats
- ☐ Sheep
- ☐ Chicken or other poultry
- ☐ None of the above

20. a. How many cows?

____

20. b. How many horses, donkeys or mules?

____

20. c. How many goats?

____

20. d. How many sheep?

____

20. e. How many chickens or other poultry?

____

21. Main material of floor

- ◯ Natural - Earth/Sand
- ◯ Natural - Dung
- ◯ Rudimentary - Wood planks
- ◯ Rudimentary - Palm/bamboo
- ◯ Finished - Parquet or polished wood
- ◯ Finished - Vinyl or asphalt strips
- ◯ Finished - Ceramic tiles
- ◯ Finished - Cement
- ◯ Finished - Carpet
- ◯ Other

21. b. Other material of floor

____

22. Main material of roof

- ◯ No roof
- ◯ Natural - Thatch/palm leaf
- ◯ Natural - Sod
- ◯ Rudimentary - Rustic mat
- ◯ Rudimentary - Palm/bamboo
- ◯ Rudimentary - Wood planks
- ◯ Rudimentary - Cardboard
- ◯ Finished - Metal (including zinc)
- ◯ Finished - Wood
- ◯ Finished - Calamine/cement fiber
- ◯ Finished - Ceramic tiles
- ◯ Finished - Cement
- ◯ Finished - Roofing shingles
- ◯ Other

22. b. Other material of roof

____

23. Main material of exterior walls

- ○ No walls
- ○ Natural - No walls
- ○ Natural - Cane/palm/trunks
- ○ Natural - Dirt
- ○ Rudimentary - Bamboo with mud
- ○ Rudimentary - Stone with mud
- ○ Rudimentary - Uncovered adobe
- ○ Rudimentary - Plywood
- ○ Rudimentary - Cardboard
- ○ Rudimentary - Reused wood
- ○ Finished walls - Cement
- ○ Finished walls - Stone with lime/cement
- ○ Finished walls - Bricks
- ○ Finished walls - Cement blocks
- ○ Finished walls - Covere adobe
- ○ Finished walls - Wood planks/shingles
- ○ Other

23. b. Other material of exterior walls

___

24. Does any member of your household live outside of Guyana?

- ○ Yes
- ○ No

24. b. Where?

___

24. c. What is your relation to the person(s) living outside Guyana?

- ☐ Spouse
- ☐ Son/Daughter
- ☐ Brother/Sister
- ☐ Mother/Father
- ☐ Uncle/Aunt
- ☐ Cousin
- ☐ Friend
- ☐ Other

24. d. Other relation

____

25. How many workers in total work in this place (including temporaray workers)?

____

26. What are the main activities of the place?

- [ ] Sugar
- [ ] Gold
- [ ] Bauxite
- [ ] Agriculture
- [ ] Restaurant
- [ ] Shop
- [ ] Other

26. b. Other main activity

____

27. Type of school

- ○ Kindergarten
- ○ Primary
- ○ Secondary
- ○ University
- ○ Other

28. How many students are there in this institution?

____

29. Type of health facility

- ○ Dispensary/Health Post
- ○ Health Centre
- ○ Hospital
- ○ Private clinic

30. How many outpatients visits do you have on an ordinary day in total?

____

31. How many beds are there in the hospital?

____

32. Which is the disease you fear the most?

____

33. Do you know what the Zika virus is?

- ◯ Yes
- ◯ No

33. a. Can you briefly describe what you know about the Zika virus?

- ☐ Mosquito(es)
- ☐ Fever
- ☐ Skin rash
- ☐ Pregnancy
- ☐ Microcephaly
- ☐ Paralisis

34. Do you know what Dengue fever is?

- ◯ Yes
- ◯ No

34. b. Can you briefly describe what you know about Dengue fever?

- ☐ Mosquito(es)
- ☐ Fever
- ☐ Skin rash

35. Do you know what Malaria is?

- ◯ Yes
- ◯ No

35. b. Can you briefly describe what you know about Malaria?

- ☐ Mosquito(es)
- ☐ Fever
- ☐ Headache
- ☐ Cold sweat
- ☐ Vivax
- ☐ Falciparum

36. Do you know what Bush Yaws (Leishmaniasis) is?

- ◯ Yes
- ◯ No

36. a. Can you briefly describe what you know about Bush Yaws (Leishmaniasis)?

- [ ] Sandfly
- [ ] Skin lesion
- [ ] Dog

37. How much do you think you and the people in this place are at risk of Zika virus on a scale from 0 to 10 (0 - risk; 10 - very high risk)?

____

38. How much do you think you and the people in this place are at risk of Dengue on a scale from 0 to 10 (0 - risk; 10 - very high risk)?

____

39. How much do you think you and the people in this place are at risk of Malaria on a scale from 0 to 10 (0 - risk; 10 - very high risk)?

____

40. How much do you think you and the people in this place are at risk of Bush Yaws (Leishmaniasis) on a scale from 0 to 10 (0 - risk; 10 - very high risk)?

____

**41. In 5 years, what impact do you think the Zika virus will have on the health of the people of this community?**	Decrease	Remain the same	Increase	Don't know
**Zika virus**	○	○	○	○
**42. In 5 years, what impact do you think the Dengue fever will have on the health of the people of this community?**	Decrease	Remain the same	Increase	Don't know
**Dengue fever**	○	○	○	○
**43. In 5 years, what impact do you think Malaria will have on the health of the people of this community?**	Decrease	Remain the same	Increase	Don't know
**Malaria**	○	○	○	○
**44. In 5 years, what impact do you think Bush Yaws (Leishmaniasis) will have on the health of the people of this community?**	Decrease	Remain the same	Increase	Don't know
**Bush Yaws (Leishmaniasis)**	○	○	○	○

45. What would you do to avoid the Zika virus? (Multiple replies allowed)

- [ ] Nothing
- [ ] Screened windows
- [ ] Skin repellent
- [ ] Mosquito zapper racket
- [ ] Beeper mosquito
- [ ] Fogging
- [ ] Indoor residual spray
- [ ] Mosquito coils
- [ ] Bed nets
- [ ] Bracelets
- [ ] Sitting next to a fire

45. What would you do to avoid the Dengue fever? (Multiple replies allowed)

- [ ] Nothing
- [ ] Screened windows
- [ ] Skin repellent
- [ ] Mosquito zapper racket
- [ ] Beeper mosquito
- [ ] Fogging
- [ ] Indoor residual spray
- [ ] Mosquito coils
- [ ] Bed nets
- [ ] Bracelets
- [ ] Sitting next to a fire

45. What would you do to avoid Malaria? (Multiple replies allowed)

- [ ] Nothing
- [ ] Screened windows
- [ ] Skin repellent
- [ ] Mosquito zapper racket
- [ ] Beeper mosquito
- [ ] Fogging
- [ ] Indoor residual spray
- [ ] Mosquito coils
- [ ] Bed nets
- [ ] Bracelets
- [ ] Sitting next to a fire

45. What would you do to avoid Bush Yaws (Leishmaniasis? (Multiple replies allowed)

- [ ] Nothing
- [ ] Screened windows
- [ ] Skin repellent
- [ ] Mosquito zapper racket
- [ ] Beeper mosquito
- [ ] Fogging
- [ ] Indoor residual spray
- [ ] Mosquito coils
- [ ] Bed nets
- [ ] Bracelets
- [ ] Sitting next to a fire

46. How effective do you perceive the preventative measures you would use on a scale from 0 to 10?

____

SHOW INTERVIEWEE EMB1 PICTURE

Haga clic aquí para subir el archivo. (<5MB)

SHOWINTERVIEWEE EMB2 PICTURE

Haga clic aquí para subir el archivo. (<5MB)

SHOW INTERVIEWEE EMB3 PICTURE

Haga clic aquí para subir el archivo. (<5MB)

SWITCH TO THE RANDOM NUMBER GENERATOR APP TO GET A NUMBER FROM 1 TO 13

Enter the number you would have generated

- ○ 1
- ○ 2
- ○ 3
- ○ 4
- ○ 5
- ○ 6
- ○ 7
- ○ 8
- ○ 9
- ○ 10
- ○ 11
- ○ 12
- ○ 13

SHOW THE CORRESPONDING PICTURE NUMBER TO THE NUMBER YOU GENERATED

Haga clic aquí para subir el archivo. (<5MB)

47. a. In your opinion, which of the three options described do you think is best?

- ○ EMB1
- ○ EMB2
- ○ EMB3

47. b. Taking into account your circumstances, which one of the options would you take?

- ○ EMB1
- ○ EMB2
- ○ EMB3
- ○ None
